# Prevalence of pulmonary artery dilation in non-cystic fibrosis bronchiectasis: A CT analysis from a cohort of the US Bronchiectasis and Nontuberculous Mycobacteria Research Registry

**DOI:** 10.21203/rs.3.rs-2711488/v1

**Published:** 2023-03-22

**Authors:** Elham Karamooz, Anupama G. Brixey, Chara E. Rydzak, Steven L. Primack, Sheila Markwardt, Alan F. Barker

**Affiliations:** Oregon Health & Science University Pulmonary & Critical Care; Cardiothoracic Imaging Section, Department of Diagnostic Radiology, Oregon Health & Science University; Cardiothoracic Imaging Section, Department of Diagnostic Radiology, Oregon Health & Science University; Cardiothoracic Imaging Section, Department of Diagnostic Radiology, Oregon Health & Science University; Oregon Health & Science University-Portland State University School of Public Health; Oregon Health & Science University Pulmonary & Critical Care

**Keywords:** Bronchial Diseases, nontuberculous mycobacteria, pulmonary artery, hypoxemia

## Abstract

Although pulmonary artery (PA) dilation is independently associated with significant morbidity and mortality in patients with pulmonary diseases irrespective of diagnosed pulmonary hypertension, its relationship to nontuberculous mycobacteria (NTM) is unknown. To determine the prevalence of PA dilation in patients with NTM-predominant non-CF bronchiectasis, we evaluated the chest computed tomography (CT) scans from 321 patient in the United States based Bronchiectasis and NTM Research Registry. The majority of our cohort had NTM infection. We measured the severity of bronchiectasis using modified Reiff criteria and measured the diameters of the PA and aorta (Ao), with PA dilation defined as a PA:Ao ratio > 0.9. Forty-two patients (13%) were found to have PA dilation. PA dilation was positively associated with the use of supplemental oxygen (*p* < 0.001), but there was no association between PA dilation and NTM infection.

## Introduction

Bronchiectasis is defined on imaging as bronchial dilation greater than 1.5x the adjacent artery [1]. Chronic inflammation and infection can lead to subsequent parenchymal destruction [2]. Destruction of alveolar units causes hypoxia, which results in pulmonary vasoconstriction and eventually pulmonary hypertension [3]. Pulmonary hypertension can manifest as pulmonary artery (PA) dilation, and PA dilation can be identified on chest computed tomography (CT) by the presence of a PA ≥ 29 mm in diameter in men and ≥ 27 mm in diameter in women or a ratio of the PA to aorta (Ao) > 0.9 [4]. The clinical relevance of PA dilation has been well established. For example, in severe chronic obstructive pulmonary disease (COPD), the prevalence of PA dilation ranges from 20–24% [5, 6], is positively associated with exacerbations and mortality [6–8], and the presence of a PA:Ao ratio > 1 on chest CT outperformed echocardiography in predicting elevated mean pulmonary artery pressure [9]. In a study of 91 patients with bronchiectasis, the majority of whom had idiopathic bronchiectasis, an average diameter of the right and left main Pas > 18 mm was a predictor of increased mortality with a hazard ratio of 1.24 [10]. Thus far, there have been no studies examining the prevalence of PA dilation in nontuberculous mycobacteria (NTM) predominant non-cystic fibrosis (CF) bronchiectasis.

The Bronchiectasis and NTM Research Registry (BRR) was created by the COPD Foundation to foster research in non-CF bronchiectasis and NTM lung disease [11]. The majority of patients with non-CF bronchiectasis at Oregon Health & Science University (OHSU) have NTM infections. NTM infection is associated with increased mortality [12, 13] with data demonstrating that pulmonary hypertension is associated with increased mortality in patients with NTM lung disease [14]. However, the prevalence of PA dilation in NTM is unknown. The objective of this study was to measure the prevalence of PA dilation in a large cohort with non-CF bronchiectasis predominantly due to NTM. The association of the following factors with PA dilation was also evaluated: supplemental oxygen use, severity of bronchiectasis, tobacco use, and NTM in the sputum culture.

## Methods

### Study population.

We identified 380 patients at OHSU participating in the BRR from 2010–2019. We excluded 6 patients with duplicate records, 17 patients without CT scans available for review, and 1 patient who had not completed enrollment in the BRR. Of the remaining 356 patients, CT chest performed in closest proximity to enrollment date in the BRR was reviewed for each patient. An additional 35 patients were excluded due to poor quality CT scans limiting evaluation (n=2), presence of traction bronchiectasis as a result of idiopathic interstitial pneumonia-related pulmonary fibrosis (n=3), diagnosis of CF after enrollment (n=3), and an inability to score one or more lobes due to lobectomy/resection or significant atelectasis/scarring that precluded scoring (n=27). This left a total of 321 patients in the cross-sectional analysis. The clinical variables of supplemental oxygen use, tobacco use, and results of the sputum culture were collected from the BRR and electronic medical record.

### Modified Reiff Score and PA:Ao.

One of three fellowship-trained thoracic radiologists reviewed and scored every CT scan of the chest using modified Reiff criteria [15,16], with agreement on how to obtain the PA and Ao measurements made amongst the three radiologists prior to performance of all measurements. The main PA and Ao diameter were measured on axial soft tissue images with electronic calipers at the level of the bifurcation of the right main pulmonary artery [4], with elevation defined as PA:Ao > 0.9. The radiologists were blinded to all clinical data and CT scans were reviewed in batches. Each lobe, including the lingula, was scored based on the highest severity of airway dilation: no bronchiectasis (score=0), <2x diameter of adjacent pulmonary artery (score=1), 2–3x diameter of adjacent pulmonary artery (score=2), >3x diameter of adjacent pulmonary artery (score=3). A total score of 0 is normal, 1–6 is mild bronchiectasis, 7–12 is moderate bronchiectasis and 13–18 is severe bronchiectasis.

### Statistical Analysis.

We used ANOVA for categorical independent variables, t-tests for binary independent variables, and linear regression to determine the association between PA:Ao ratio and modified Reiff score. Post-hoc, pair-wise differences were estimated to determine which categorical factor means were different from each other; multiple comparisons were adjusted for using the Tukey-Kramer method. All model assumptions were assessed. All analyses were performed in STATA/SE 15.1 (StataCorp, College Station, TX).

## Results

A total of 321 patients were included in the final analysis (Table 1). The mean age was 67 years and 83% were female. Pulmonary function testing revealed moderate obstructive lung disease based on GOLD criteria, with a mean FEV1% of 73.5%. In our cohort, 9% of patients required the use of supplemental oxygen and < 4% of patients were active smokers. Sputum culture data were available for 184 patients. Sputum cultures were positive for NTM in 75% of the registry patients (58% were only positive for NTM and 17% were positive for NTM and *Pseudomonas aeruginosa*). Measurements of bronchiectasis severity showed that 89% had mild to moderate disease (Table 2). PA measurements revealed a mean PA diameter of 24.6 mm (SD 3.5 mm) with a mean PA:Ao ratio of 0.79 (SD 0.11). A total of 42 out of 321 patients (13.1%) demonstrated PA dilation as defined by PA:Ao ratio > 0.9.

Prespecified factors were evaluated to identify associations with PA dilation. The factors evaluated were use of supplemental oxygen, severity of bronchiectasis, tobacco use, and microbial composition of sputum culture. There was a statistically significant association between PA dilation and use of supplemental oxygen compared to non-users: the mean PA:Ao was 0.87 (SD ± 0.11) for those using supplemental oxygen compared to mean PA:Ao of 0.78 (SD ± 0.11) among non-users (95% CI 0.03–0.11, *p* < 0.001) (Table 3). There was no statistically significant association between presence or absence of PA dilation and severity of bronchiectasis, tobacco use, or NTM infection.

## Discussion

In this report, we analyzed CT scans from 321 patients with non-CF bronchiectasis in order to determine the prevalence of PA dilation within this cohort and to evaluate whether prespecified factors—supplemental oxygen use, severity of bronchiectasis, tobacco use, or NTM in the sputum culture—were associated with PA dilation. These factors were chosen based on their association with hypoxemia and/or parenchymal destruction, and therefore, their ability to induce PA dilation. Overall, we found that 13% of the cohort met criteria for PA dilation. Of the factors examined, only use of supplemental oxygen was associated with PA dilation. This result is consistent with studies showing an association between hypoxemia and pulmonary hypertension in patients with bronchiectasis [17, 18]. We used descriptive statistics in our study and we did not find any association between NTM and PA dilation; prospective studies are needed to better delineate contributions from various factors to the PA dilation in patients with NTM and non-CF bronchiectasis.

The prevalence of PA dilation in our cohort is lower than the prevalence reported in patients with moderate-severe COPD [5, 6]. One explanation for this is that our cohort had mild-moderate bronchiectasis, and therefore, had less parenchymal and vascular destruction. A lower level of parenchymal and vascular destruction is less likely to lead to PA dilation and this is supported by the modest prevalence of hypoxemia in the cohort, with 9% using supplemental oxygen. To measure the severity of bronchiectasis, we used the modified Reiff score because of its ease of use despite differences in CT techniques. In our cohort, the modified Reiff score found higher scores in the middle lobe and lingula (data not shown), which is a distribution of disease that is consistent with the overall BRR data [11]. This consistency supports the accuracy of the modified Reiff scoring in our study.

Limitations of this study include the single center design. This was necessary because the BRR does not have a central imaging repository. Also, the BRR does not include right heart catheterization data and very few patients have echocardiograms, making it difficult to determine the correlation between PA dilation and mean pulmonary artery pressure. However, previous work in COPD found PA dilation outperformed echocardiography in detecting PH [9]. An additional limitation is that follow-up CT scans were not included in this analysis and thus data are lacking regarding the development of PA dilation over time. Despite these limitations, our analysis included a large number of patients and our study provides important additional data on the low prevalence of PA dilation in a cohort of patients with NTM predominant infection and mild-moderate non-CF bronchiectasis. Furthermore, PA dilation in this cohort was associated only with use of supplemental oxygen, suggesting that further work-up for underlying pulmonary hypertension should be implemented in patients non-CF bronchiectasis that require supplemental oxygen.

## Figures and Tables

**Table 1:** Baseline demographics and clinical characteristics

Factor	Value

Age, mean (SD), N= 321	67.3 (13.2)

Gender, N= 321	
• Female #	267 (83.2%)
• Male #	54 (16.8%)

# of exacerbations in the last 2 years, N= 261	
• 0 #	138 (52.9%)
• 1–2 #	81 (31.0%)
• 3+ #	42 (16.1%)

# of exacerbations in the last 2 years, mean (SD)	1. (1.5)

Tobacco Use, N= 312	
• Non-user #	175 (56.1%)
• Former user #	125 (40.1%)
• Current user #	12 (3.8%)

Oxygen use, N= 310	
• No #	282 (91.0%)
• Yes #	28 (9.0%)

FEV1 (L), mean (SD), N= 184	1.91 (0.72)

FEV1%, mean (SD), N= 182	73.5 (23.2)

FEV1/FVC%, mean (SD), N= 182	66.8 (11.1)

Bacterial culture, N= 184	
• NTM #	106 (57.6%)
• Pseudomonas #	23 (12.5%)
• NTM & Pseudomonas #	31 (16.8%)
• Neither NTM nor Pseudomonas #	24 (13.0 %)

Abbreviations: FEV1, forced expiratory volume in 1 s; FVC, forced vital capacity; NTM, nontuberculous mycobacteria.

**Table 2:** CT measurements of pulmonary artery, aorta and bronchiectasis severity

Factor	Value

Modified Reiff Score, mean (SD), N=321	7.1 (3.8)

Modified Reiff Score, N=321^a^	
• Normal (score= 0)	7 (2.2%)
• Mild (score= 1–6)	143 (44.5%)
• Moderate (score= 7–12)	143 (44.5%)
• Severe (score= 13–18)	28 (8.7%)

Ao diameter (mm), mean (SD), N=321	31.5 (4.0)

PA diameter (mm), mean (SD), N=321^b^	24.6 (3.5)

PA/Ao, mean (SD), N=321	0.79 (0.11)

Abbreviations: PA, pulmonary artery; Ao, aorta.

a 27 CT scans had 1 or more lobes that could not be scored because of lobectomy/resection, atelectasis, or scarring. Those scans were not included in any analysis.

b 66 CT scans were contrast enhanced.

**Table 3:** Clinical factors associated with pulmonary artery dilation

Clinical Factor	Mean PA/Ao (SD)	*p*-value

Oxygen use N= 310		<0.001^a^
• No, #282	0.78 (0.11)	
• Yes, #28	0.87 (0.11)	

Severity of bronchiectasis (modified Reiff score)	Not applicable	0.07^b^

Tobacco use N= 312		0.12^c^
• Non-user, #175	0.78 (0.11)	
• Former user, #125	0.80 (0.11)	
• Current user, #12	0.81 (0.13)	

Sputum culture N= 184		0.56^c^
• NTM, #106	0.79 (0.11)	
• Pseudomonas, #23	0.82 (0.09)	
• NTM and Pseudomonas, #31	0.81 (0.12)	
• Neither NTM nor Pseudomonas, #24	0.79 (0.10)	

Abbreviations: NTM, nontuberculous mycobacteria.

a *p*-value from *t*-test

b *p*-value from regression of modified Reiff score on PA/Ao. The β-coefficient from the regression was 0.003 (for every 1 point increase in modified Reiff score, the PA/Ao increases by 0.003 units).

c *p*-value from ANOVA F-statistic.
